# OA08.03. Electroacupuncture alleviates hyperalgesia by inhibiting spinal interleukin-17 in an inflammatory pain rat model

**DOI:** 10.1186/1472-6882-12-S1-O31

**Published:** 2012-06-12

**Authors:** R Zhang, X Meng, Y Zhang, X Shen, A Li, B Berman, K Ren, P Wei, L Lao

**Affiliations:** 1 Center for Integrative Medicine, Baltimore, USA; 2 Department of Neurobiology, Shanxi Medical University, Taiyuan, China; 3 Shanghai University of Traditional Chinese Medicine, Shanghai, China; 4 Dept. of Neural and Pain Sciences, Dental Sch., Univ. of Maryland, Baltimore, USA

## Purpose

Previous studies demonstrated that electroacupuncture (EA) alleviates hyperalgesia, but the mechanisms remained unclear. Because it is well known that interleukin-17 (IL-17) is associated with autoimmune disorders, the present study was designed to determine whether spinal IL-17 plays a role in inflammatory pain and, if so, whether EA inhibits spinal IL-17 expression during such pain.

## Methods

Hyperalgesia was induced by injecting complete Freund’s adjuvant (CFA, 0.08 ml, 40 μg Mycobacterium tuberculosis) into one hind paw of each rat. EA treatment, 10 Hz at 3 mA, was given at acupoint GB30 twice for 20 min each, once immediately post-CFA and again 2 hours later. Paw withdrawal latency (PWL) was tested before (-48 h) and 2 and 24 hours after CFA to assess behavioral hyperalgesia. IL-17 antibody (0.2-2 µg/rat) was given intrathecally (i.t.) 24 h before CFA to block the action of basal IL-17 and 2 hours prior to each of two PWL tests to block CFA-induced IL-17. I.t. recombinant IL-17 (10-400 ng/rat) was administered to naive rats to determine its effects on PWL and phosphorylation of NR1 (p-NR1). P-NR1 is known to modulate N-methyl-D-aspartate receptor (NMDAR) activity and to facilitate pain. Spinal cords were removed for immunostaining of IL-17, double immunostaining of IL-17/cell markers and IL-17 receptor subtype A (IL-17RA)/NR1, and western blot to measure p-NR1 and IL-17RA.

## Results

The data showed that (1) IL-17 is selectively up-regulated in astrocytes, 2) IL-17RA is localized and up-regulated in NR1-immunoreactive neurons, and 3) an IL-17 antibody at 2 µg/rat significantly increased PWL (p<0.05) and decreased p-NR1 and IL-17RA in CFA- and IL-17-injected rats compared to control. EA significantly inhibited hyperalgesia, IL-17, IL-17RA, and p-NR1.

## Conclusion

The results suggest that (1) spinal IL-17 is produced by astrocytes and enhances p-NR1 to facilitate inflammatory pain, and 2) EA inhibits hyperalgesia by suppressing IL-17.

